# Oligomerization of optically active *N*-(4-hydroxyphenyl)mandelamide in the presence of β-cyclodextrin and the minor role of chirality

**DOI:** 10.3762/bjoc.10.246

**Published:** 2014-10-10

**Authors:** Helmut Ritter, Antonia Stöhr, Philippe Favresse

**Affiliations:** 1 Institute of Organic Chemistry and Macromolecular Chemistry, Heinrich-Heine-University Düsseldorf, Universitätsstraße 1, Düsseldorf, 40225, Germany; 2 Coatings & Additives, Evonik Industries AG, Goldschmidtstraße 100, Essen, 45127, Germany

**Keywords:** cyclodextrin, enantioselectivity, enzyme, oxidative coupling, phenol oligomer

## Abstract

The oxidative oligomerization of a chiral mandelamide derivative (*N*-(4-hydroxyphenyl)mandelamide, **1**) was performed in the presence of horseradish peroxidase, laccase and *N,N'*-bis(salicylidene)ethylenediamine-iron(II) to obtain chiral oligophenols **2**. The low enantioselectivity of the enzymatic catalyzed asymmetric enantiomer-differentiating oligomerizations was investigated. In addition, the poor influence of cyclodextrin on the enantioselectivity of enzymatic catalyzed asymmetric enantiomer-differentiating oligomerizations was studied.

## Introduction

Lignin consists of mechanical stabilizing polyphenols and thus plays an important role in many plants [1–2]. For the in vitro synthesis of polyphenols via oxidative coupling reactions, laccases and peroxidases are suitable enzymes to catalyze the oligomerization of substituted electron-rich phenols in the presence of oxidizing agents [3–4]. In addition to that, *N,N'*-bis(salicylidene)ethylenediamine-iron(II) (iron(II)-salen) represents an alternative catalyst for oxidative coupling reactions of phenol derivatives [5]. The use of β-cyclodextrin (CD) allows the oxidative coupling of poor water soluble phenol derivatives via complexation without using of organic solvents [6–8]. However, to the best of our knowledge, there were no studies published dealing with the potential enantioselective control of enzyme catalyzed oligomerization reaction of chiral phenol derivatives, respectively. Thus, the enantioselectivity of enzymatic asymmetric enantiomer-differentiating oligomerizations of a chiral mandeloamide-phenol derivative as model compound and the influence of cyclodextrin is a main subject of the present study.

## Results and Discussion

**Scheme 1:**
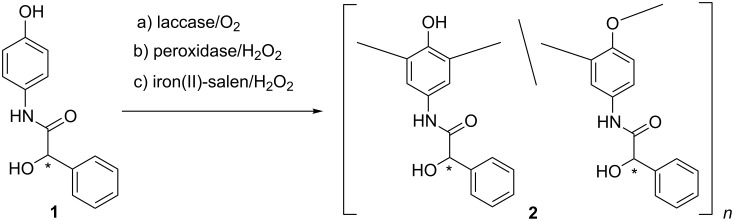
Oligomerization of *N*-(4-hydroxyphenyl)mandelamide (**1**).

The chiral *N*-(4-hydroxyphenyl)mandelamide (**1**) was synthesized through condensation of *p*-aminophenol with (*R*)*-* or (*S*)*-*mandelic acid, respectively in presence of dicyclohexylcarbodiimide as condensing agent. For oligomerization of **1** via oxidative coupling laccase from *Pleurotus ostreatus*, peroxidase from horseradish or iron(II)-salen were used as catalysts. The obtained yellow powdery oligomers **2** show high solubility in many commonly used organic solvents like acetone, THF, ethanol, methanol, acetonitrile and 1,4-dioxan. Because of the broad signals of the oligomers **2** in the ^1^H NMR spectra the ratio of the phenylene and oxyphenylene units (Scheme 1) could not be clearly determined.

**Figure 1:**
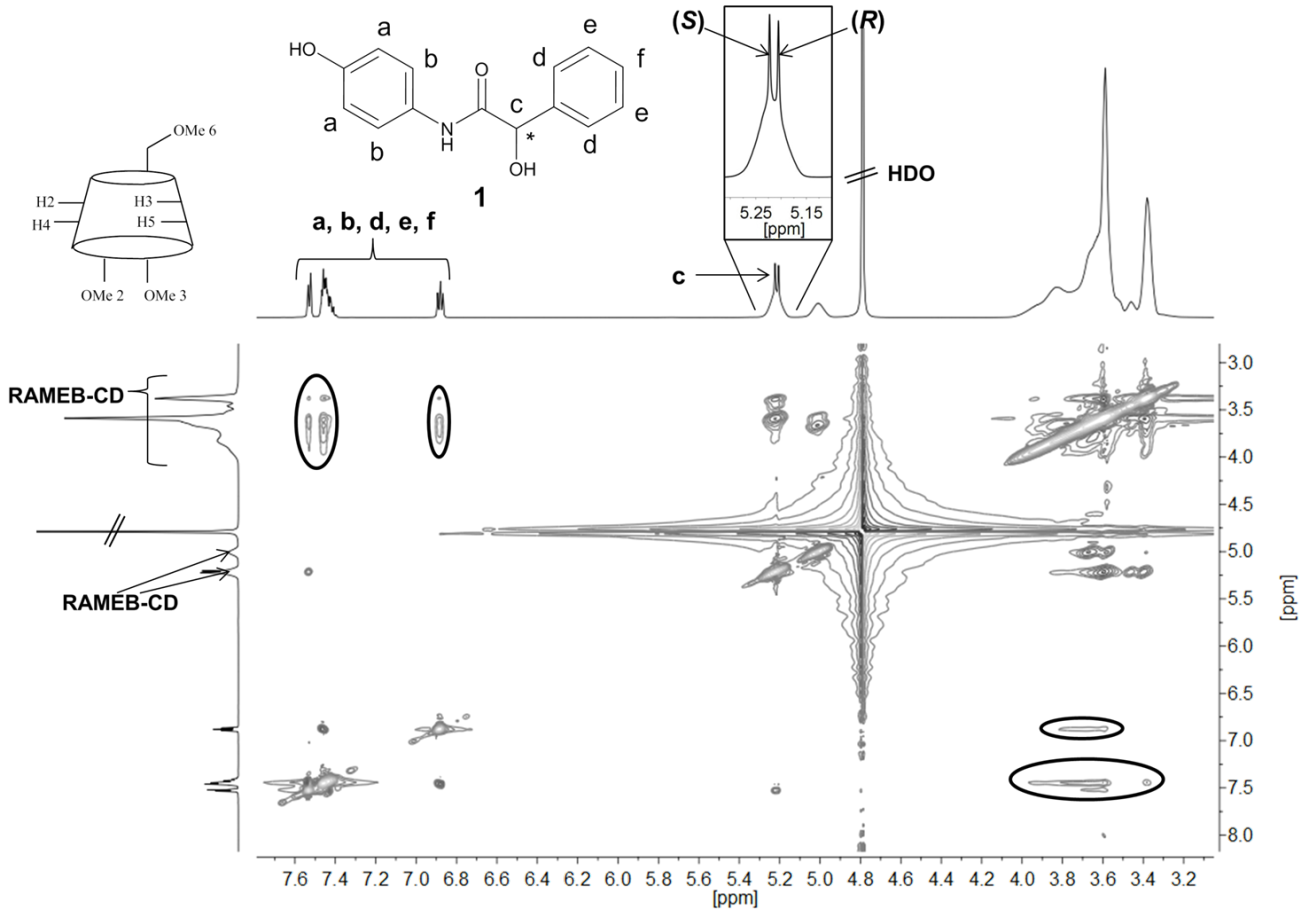
2D ROESY NMR spectrum (600 MHz, D_2_O) of the racemate **1** complexed with RAMEB-CD.

The oligomerization of **1** in water could be easily performed through complexation of the monomer **1** with randomly methylated β-cyclodextrin (RAMEB-CD). The formation of the complex was verified with 2D ROESY NMR spectroscopy. The magnetic interaction of the monomer with the cavity of RAMEB-CD is obvious in the 2D ROESY NMR spectra as shown in Figure 1 (marked areas). Principally, cyclodextrins and their derivatives are able to discriminate enantiomeric compounds [9–10]. Such chirality recognition is provable with ^1^H NMR spectroscopy because of the different induced shift of the protons which became diastereotopic through complexation [11–12]. Actually, the chirality discrimination of **1** with RAMEB-CD is evident from the different induced shift of the protons 8 at 5.2 ppm (zoomed out in Figure 1).

**Figure 2:**
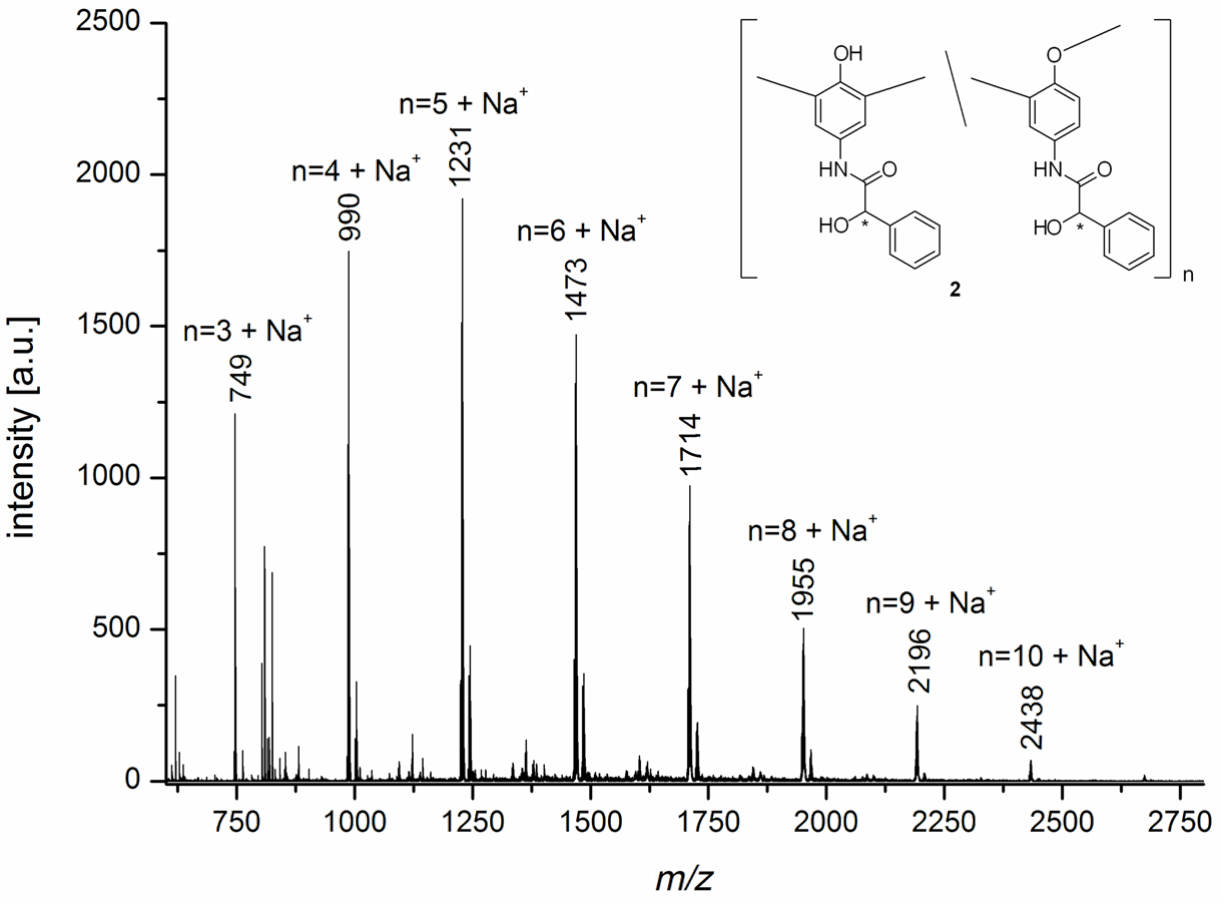
MALDI–TOF MS spectrum of the oligomers synthesized with *laccase* from the racemate **1**.

The MALDI–TOF MS measurements indicate the formation of oligomers **2** from the monomer **1** as shown in Figure 2. As expected the repetitive unit has a molecular mass of 241 g/mol, which confirms the linkage of the monomers via a formal abstraction of two hydrogen atoms. The highest molecular weight oligomers **2** obtained through enzymatic oligomerization consists of up to 10 repetitive units which could be detected by MALDI–TOF MS measurements. Furthermore comparable molecular weights are accessible through oligomerization of **1** with iron(II)-salen as catalyst. Here oligomers **2** with up to 8 repetitive units are detectable.

The conversion of the enantiomers of **1** during the enzymatic oligomerization has been studied using chiral HPLC. Accordingly, the racemate of **1** was oligomerized three times with each enzyme in the absence of RAMEB-CD or in the presence of RAMEB-CD, respectively to evaluate the reproducibility. The isolated monomeric residual of each oligomerization was measured twice. The obtained enantiomeric excess (ee) values of the monomeric residual are given in Table 1. Because of the rapid conversion of the monomer **1** during the oligomerization with highly active peroxidase–H_2_O_2_ system at room temperature, the reaction time was limited to one minute at 0 °C. In the presence of the lower active laccase–O_2_ system, the reaction was carried out for 4 h at room temperature.

**Table 1:** Enantiomeric excess values (ee) of the monomeric residual of the enzymatic oligomerization of **1**.

used enzyme, use of CD	ee (%)^a^

laccase	racemic mixture
peroxidase	6 *S*
laccase + CD	4 *R*
peroxidase + CD	8 *R*

^a^Calculated on the basis of the surfaces of chiral HPLC peaks, enantiomers were separated from the reaction mixture by column chromatography with ethylacetate/*n*-hexane (2:1) as eluent.

In the absence of RAMEB-CD it is apparent that laccase shows no enantioselectivity. However it can be established that during the oligomerization with peroxidase the (*S*)-enantiomer **1** slightly enriches the reaction solution. Additionally to that, it was of some interest to verify, whether the complexation of the enantiomers with RAMEB-CD affects the conversion of the enantiomers. Therefore, the relatively slow oligomerizations in the presence of laccase were carried out in pH 5 buffer at room temperature for 4 hours. The rapid oligomerizations in the presence of peroxidase were carried out in pH 7 buffer at 0 °C for 1 min. It was found that, in the presence of RAMEB-CD, the (*R*)-enantiomer of **1** slightly enriches the reaction mixture with laccase as well as with peroxidase. As already mentioned above, the opposite effect was observed when the oligomerization was carried out with peroxidase without using RAMEB-CD. However, the obtained data show that the degree of enantioselectivity during conversion of **1** is generally very low.

## Conclusion

Oligomers of both enantiomers of *N*-(4-hydroxyphenyl)mandelamide (**1**) were obtained though oxidative coupling with peroxidase and laccase and also via oligomerization with iron(II)-salen and hydrogen peroxide as a catalyzing system. In the presence of RAMEB-CD it was possible to oligomerize the poorly water soluble enantiomers of **1** without using organic solvents. In the case of oligomerization with laccase the monomeric residual keeps racemic. In contrast to this, with peroxidase the (*S*)-enantiomer of **1** slightly enriches the monomeric residual. RAMEB-CD promotes the (*S*)-enantiomer of **1** which was more readily converted with both enzymes. However this preference is very low in all cases.

## Experimental

### Materials

Horseradish peroxidase practical grade I (370,2 U/mg) was purchased from Appli Chem and Laccase from pleurotus ostreatus (9,4 U/mg) from Sigma-Aldrich. *p*-Aminophenol was bought from Grüssing, *N,N'*-dicyclohexylcarbodiimide from Appli Chem, *N*-hydroxysuccinimide from Fluka and RAMEB-CD from Wacker Chemie AG. Racemic- and (*S*)-mandelic acid was obtained from Merck and (*R*)-mandelic acid from TCI. The used buffers were ordered from Carl Roth. The solvents used for synthesis were used in p.a. quality. Technical solvents used for column chromatography were distilled before usage.

### Measurements

The 300 MHz ^1^H and the 75 MHz ^13^C-{^1^H} NMR spectra were measured on a Bruker AVIII 300 and the 600 MHz ^1^H NMR spectra were performed using a Bruker Advance III 600. The 500 MHz ^1^H NMR spectra were obtained using a Bruker AV DRX 500. MALDI–TOF spectra were performed on a Bruker Ultraflex TOF mass spectrometer, the molecular masses being recorded in linear mode. Dithranol was used as a matrix and sodium trifluoroacetate (NaTFA) as ionization reagent. The samples were dissolved in DMF. DSC measurements were performed using a Mettler Toledo DSC 822. The GC–MS analyses were carried out on Thermo Finnigan Trace DSQ GC/MS-System with THF as solvent. HPLC analyses were performed on BioTek Konton 525 equipped with UV detector (at 260 nm, diode array detector HPLC 540) at room temperature (column: Chiralcel OD-H, eluent: methanol/water 8:2, flow rate: 0,4 mL/min). Optical rotations were measured with a Perkin-Elmer 341 polarimeter in THF as solvent, with a concentration of 10 mg ml^−1^. GPC analysis were carried out at 60 °C with *N,N*-dimethylformamide as eluent with a flow rate of 1 mL/min using a ViscotekGPCmax VE2001 system and a Viscotek VE 3500 RI detector. The system was calibrated with polystyrene standards with a molecular range from 1,280 g/mol to 1,373,000 g/mol.

### Syntheses

#### Synthesis of *N*-(4-hydroxyphenyl)mandelamide (**1**)

7.61 g (50 mmol) Mandelic acid and 5.75 g (50 mmol) *N*-hydroxysuccinimide were dissolved in 150 mL acetone and cooled in an ice–water bath. Subsequently, a suspension of 10.32 g (50 mmol) dicyclohexylcarbodiimide in 50 mL acetone was added and the reaction mixture stirred for 2.5 hours at 0 °C. After that, 5.46 g (50 mmol) *p*-aminophenol was added and the ice bath was removed. The reaction mixture was stirred for 24 hours at room temperature. The precipitated dicyclohexylurea was filtered off. Then the solution was concentrated under reduced pressure and the product purified by column chromatography (eluent: *n*-hexane/ethyl acetate 1:2). Yield depending on the use of racemic or (*S*)-, (*R*)-enantiomer of mandelic acid: (*RS*)-**1** = 9.16 g (75%), (*S*)-**1** = 10.03 g (82%), (*R*)-**1** = 9.63 g (79%). mp: (*RS*)-**1**: 97 °C, (*S*)-**1**: 155 °C, (*R*)-**1**: 153 °C; optical rotation (THF): (*S*)-**1**: [α]_D_^20^ −3,9°, (*R*)-**1**: [α]_D_^20^ +3,4°; (*RS*)-**1**: GC–EIMS, *m*/*z*: 243 [M(**1**)]^+^, 137, 109, 79. ^1^H NMR (500 MHz, DMSO-*d*_6_) δ 9.66 (s, 1H, -NH), 9.20 (s, 1H, -OH), 7.50 (d, 2H, -ArH), 7.46 (d, 2H, -ArH), 7.35 (t, 2H, -ArH), 7.28 (t, 1H, -ArH), 6.68 (d, 2H, -ArH), 6.35 (d, 1H, -OH), 5,055 (d, 1H, -CH) ppm; ^13^C NMR (75 MHz, DMSO-*d*_6_) δ 170.42 (C=O), 153.52 (C-OH), 141.10, 130.17, 128.04, 127.51, 126.56, 121.39, 114.96 (Ar-C), 73.91 (C-OH) ppm.

#### Synthesis of oligo (*N*-(4-hydroxyphenyl)mandelamide) (**2**)

**Enzymatic oxidative oligomerization with peroxidase:** A solution of 7.5 mg peroxidase dissolved in 10 ml pH 7 buffer was added to a solution of 1.22 g (5 mmol) *N*-(4-hydroxyphenyl)mandelamide (**1**) and 40 mL 1.4-dioxan. 510 µL of hydrogen peroxide (30%) were added to the mixture in aliquots of 51 µL in 15 minutes intervals. After stirring for 2 h at room temperature, the product was precipitated by pouring into 0.5 M HCl and dried under vacuum. Yield depending on use of racemic or (*S*)-, (*R*)-enantiomer of *N*-(4-hydroxyphenyl)mandelamide (**1**): (*RS*)-**2** = 0.79 g (65%), (*S*)-**2** = 1.02 g (85%), (*R*)-**2** = 0.97 g (80%). GPC (DMF): (*RS*)-**2**: *M*_n_ = 1500 g mol^−1^, *D* = 1.22, (*S*)-**2**: *M*_n_ = 1510 g mol^−1^, *D* = 1.21, (*R*)-**2**: *M*_n_ = 1500 g mol^−1^, *D* = 1.23; (*RS*)-**2**: ^1^H NMR (300 MHz, DMSO-*d*_6_) δ 10.04–9.64 (broad signal, 1H, -NH), 7.79-6.71 (broad signal, 8H, -ArH), 6.52-6.13 (broad signal, 1H, -OH), 5.16-4.92 (broad signal, 1H, -CH) ppm.

**Enzymatic oxidative oligomerization with peroxidase in the presence of RAMEB-CD:** 1.22 g (5 mmol) *N*-(4-hydroxyphenyl)mandelamide (**1**) and 1,2 equiv RAMEB-CD were dissolved in 40 mL pH 7 buffer. Then 7.5 mg HRP dissolved in 10 mL pH 7 buffer was added to the reaction mixture. After addition of 510 µL of hydrogen peroxide (30%) in aliquots of 51 µL in 15 minutes intervals, the mixture was stirred for 2 h at room temperature. The precipitated product was isolated by filtration, washed with 0.5 M HCl, water and dried. Then the oligomer was dissolved in dioxan, acidified with HCl (37%) and stirred for 24 h. The product was precipitated in 0.5 M HCl and dried under vacuum. Yield depending on use of racemic or (*S*)-, (*R*)-enantiomer of *N*-(4-hydroxyphenyl)mandelamide (**1**): (*RS*)-**2** = 0.74 g (61%), (*S*)-**2** = 0.72 g (60%), (*R*)-**2** = 0.78 g (65%); GPC (DMF): (*RS*)-**2**: *M*_n_ = 1540 g mol^−1^, *D* = 1.19, (*S*)-**2**: *M*_n_ = 1530 g mol^−1^, *D* = 1.15, (*R*)-**2**: *M*_n_ = 1540 g mol^−1^, *D* = 1.16; (*RS*)-**2**: ^1^H NMR (300 MHz, DMSO-*d*_6_) δ 10.04–9.40 (broad signal, 1H, -NH), 7.81–6.66 (broad signal, 8H, -ArH), 6.49-6.17 (broad signal, 1H, -OH), 5.17-4.95 (broad signal, 1H, -CH) ppm.

**Enzymatic oxidative oligomerization with laccase:** To a solution of 1.22 g (5 mmol) *N*-(4-hydroxyphenyl)mandelamide (**1**) and 25 mL isopropanol a solution of 15 mg laccase and 25 mL pH 5 buffer was added. The reaction mixture was stirred for 24 h at room temperature under air. Subsequently, the precipitate was collected by filtration and dried under vacuum. Yield depending on use of racemic or (*S*)-, (*R*)-enantiomer of *N*-(4-hydroxyphenyl)mandelamide (**1**): (*RS*)-**2** = 0.91 g (75%), (*S*)-**2** = 1.03 g (85%), (*R*)-**2** = 0.95 g (79%). GPC (DMF): (*RS*)-**2**: *M*_n_ = 1680 g mol^−1^, *D* = 1.21, (*S*)-**2**: *M*_n_ = 1690 g mol^−1^, *D* = 1.21, (*R*)-**2**: *M*_n_ = 1660 g mol^−1^, *D* = 1.22; (*RS*)-**2**: ^1^H NMR (300 MHz, DMSO-*d*_6_), δ 10.09–9.41 (broad signal, 1H, -NH), 7.82–6.69 (broad signal, 8H, -ArH), 6.50−6.21 (broad signal, 1H, -OH), 5.17–4.96 (broad signal, 1H, -CH) ppm.

**Enzymatic oxidative oligomerization with laccase in the presence of RAMEB-CD:** 1.22 g (5 mmol) *N*-(4-hydroxyphenyl)mandelamide (**1**) and 1,2 equiv RAMEB-CD were dissolved in 40 ml pH 5 buffer. After addition of 15 mg laccase dissolved in 10 mL pH 5 buffer, the reaction mixture was stirred for 24 h at room temperature under air. The product was isolated by filtration then washed with 0.5 M HCl, water and died. Then the oligomer was dissolved in dioxan, acidified with HCl (37%) and stirred for 24 h. The product was precipitated in 0.5 M HCl and dried under vacuum. Yield depending on use of racemic or (*S*)-, (*R*)-enantiomer of *N*-(4-hydroxyphenyl)mandelamide (**1**): (*RS*)-**2** = 0.51 g (42%), (*S*)-**2** = 0.45 (37%), (*R*)-**2** = 0.49 g (41%). GPC (DMF): (*RS*)-**2**: *M*_n_ = 1460 g mol^−1^, *D* = 1.16, (*S*)-**2**: *M*_n_ = 1450 g mol^−1^, *D* = 1.17, (*R*)-**2**: *M*_n_ = 1420 g mol^−1^, *D* = 1.18; (*RS*)-**2**: ^1^H NMR (300 MHz, DMSO-*d*_6_) δ 10.07–9.44 (broad signal, 1H, -NH), 7.82–6.73 (broad signal, 7H, -ArH), 6.51–6.19 (broad signal, 1H, -OH), 5.15–4.97 (broad signal, 1H, -CH) ppm.

**Oxidative oligomerization with *N*,*N*'-bis(salicylidene)ethylenediamine-iron(II):** 15.6 mg (48,4 µmol) *N*, *N*´-bis(salicylidene)ethylenediamine-iron(II) was dissolved in 50 mL acetonitrile and then 1,22 g (5 mmol) *N*-(4-hydroxyphenyl)mandelamide (**1**) added. Subsequently 510 µL (5 mmol) of hydrogen peroxide (30%) was added in aliquots of 51 µL in 15 minutes intervals and the reaction mixture was stirred for 2 h at room temperature. After filtration of the reaction mixture, the product was precipitated by pouring into 0.5 M HCl and dried under vacuum. Yield: (*RS*)-**2**: 0.77 g (64%). GPC (DMF): (*RS*)-**2**: *M*_n_ = 1300 g mol^−1^, *D* = 1.15; (*RS*)-**2**: ^1^H NMR (300 MHz, DMSO-d_6_) δ 10.11–9.92 (m, 1H, -NH), 9.40–9.31 (m, 1H, -OH), 7.84–6.89 (overlapping multiplets, 7H, -ArH), 6.51–6.34 (m, 1H, -OH), 5.15–5.01 (m, 1H, -CH) ppm.
